# Supplementary material for: A cross-sectional survey of reproductive, gynecological, and breast health histories and status among people in a provincial prison for women in British Columbia
Source: PLoS One. 2026 Jan 21;21(1):e0339669. doi: 10.1371/journal.pone.0339669 (PMC12822962; doi:10.1371/journal.pone.0339669)
Supplement: Attachment — Submitted filename: Plos ONE respons to reviewers 20251118.pdf [file pone.0339669.s003.pdf]

Lines 119-121: Still says "health outcomes." Please revise for consistency.

Thank you for catching this, we have adjusted to 'histories'.

Line 291-308: It is confusing to say that your results are not representative and then state that they are! I would recommend removing the clause on lines 291-292 that reads "While this small sample is not representative of all people in provincial prisons in Canada," and just start with "The results of this survey..." Then you can condense the discussion of representation into the limitations paragraph. For that paragraph, I think it would be clearer to say something like "Although this small sample may not be representative of all people housed in women's facilities across Canada, there are some reassuring data that support representativeness within the province." And then talk about the demographic data you have for BC and the high response rate. Then the limitations about other provinces, the federal facilities, and community supervision.

We have condensed this discussion of limitations re: demographic representation on lines 297-301

Lines 301-315: Please add to the limitations some version of the text in the response to reviewers document that explains that the sample size was too small to draw comparisons between Indigenous and non-Indigenous Canadians in your sample.

Added this limitation on lines 303-305.
