# Supplementary material for: A cross-sectional survey of reproductive, gynecological, and breast health histories and status among people in a provincial prison for women in British Columbia
Source: PLoS One. 2026 Jan 21;21(1):e0339669. doi: 10.1371/journal.pone.0339669 (PMC12822962; doi:10.1371/journal.pone.0339669)
Supplement: Attachment — Submitted filename: Plos One Response to Revisions (3).docx [file pone.0339669.s002.docx]

Thank you to both reviewers for their thoughtful and helpful review of this manuscript. We have responded to each comment point by point in the table below.

| **Journal Requirements** | **Response** |
| --- | --- |
| Please ensure that your manuscript meets PLOS ONE's style requirements, including those for file naming. The PLOS ONE style templates can be found at  <https://journals.plos.org/plosone/s/file?id=wjVg/PLOSOne_formatting_sample_main_body.pdf> and  [https://journals.plos.org/plosone/s/file?id=ba62/PLOSOne_formatting_sample_title_authors_affiliations.pdf](https://track.editorialmanager.com/CL0/https:%2F%2Fjournals.plos.org%2Fplosone%2Fs%2Ffile%3Fid=ba62%2FPLOSOne_formatting_sample_title_authors_affiliations.pdf/1/010f01994bd20211-7592347d-53f7-423c-88d4-e4cc7368f7b6-000000/8cRD6cmU2namGzMnwWkTtTMFcgQ8pJpKS1AAFsse-68=228) | Thank you, we have reviewed the Style Requirements and made adjustments where necessary. |
| Please provide additional information regarding the considerations made for the prisoners included in this study. For instance, please discuss whether participants were able to opt out of the study and whether individuals who did not participate receive the same treatment offered to participants. | As this was not an intervention study, there was no treatment offered to participants. We have added additional detail with respect to participants ability to opt out of the study and statements with respect to participation not impacting participants ability to access services on line 170-181. |
| In the ethics statement in the Methods, you have specified that verbal consent was obtained. Please provide additional details regarding how this consent was documented and witnessed, and state whether this was approved by the IRB | We have provided further detail on the verbal consent process and confirmed that this process was approved by our IRB on lines 174-176. |
| We note that you have indicated that there are restrictions to data sharing for this study. For studies involving human research participant data or other sensitive data, we encourage authors to share de-identified or anonymized data. However, when data cannot be publicly shared for ethical reasons, we allow authors to make their data sets available upon request. For information on unacceptable data access restrictions, please see <http://journals.plos.org/plosone/s/data-availability#loc-unacceptable-data-access-restrictions>. If there are ethical or legal restrictions on sharing a de-identified data set, please explain them in detail (e.g., data contain potentially identifying or sensitive patient information, data are owned by a third-party organization, etc.) and who has imposed them (e.g., a Research Ethics Committee or Institutional Review Board, etc.). Please also provide contact information for a data access committee, ethics committee, or other institutional body to which data requests may be sent. Please update your Data Availability statement in the submission form accordingly. | We have updated our data availability statement to read: *Data cannot be shared publicly because of data containing potentially identifying or sensitive participant information. This restriction on data access is required by the University of New Brunswick Research Ethics Board. Data are available from the UNB Institutional Data Access / Ethics Committee for researchers who meet the criteria for access to confidential data.The Ethics Committee can be contacted at* [*ethics@unb.ca*](mailto:ethics@unb.ca)*.* |
| Please include your full ethics statement in the ‘Methods’ section of your manuscript file. In your statement, please include the full name of the IRB or ethics committee who approved or waived your study, as well as whether or not you obtained informed written or verbal consent. If consent was waived for your study, please include this information in your statement as well. | Full ethics statement including the full name of the IRB was already in the manuscript on lines 199-201. Please let us know if further information is required. |
| Reviewer 1 |  |
| Nice work that adds to the literature about health disparities in a very vulnerable population. Thank you for your willingness to do this work. | Thank you! |
| Not reported was whether there were any differences in the past histories of Indigenous and other women. This would have implications that are beyond incarcerated populations. | We are unsure if this comment is referring to the Introduction or Results.  Introduction: We have added reference to the specific barriers to reproductive healthcare faced by Indigenous women on lines 114-117.  Results: In response to both this comment and comments from Reviewer #2, we carefully considered analyzing for differences in reproductive health histories between Indigenous women and non-Indigenous women. However due to the small sample size, with slightly less than half of participants identifying as Indigenous, we have decided not to analyze for differences in Indigenous and non-Indigenous identifying participants. |
| A small point: as a limitation, you say, “Participants may not be representative of the population of people incarcerated 281 across all prisons in British Columbia, or across different provinces and territories.” Do you not have access to demographics of incarcerated women in the province? | We have added a citation to recent demographic data, which does suggest that our sample is representative (lines 304-308). We do not have access to extensive demographic data beyond age and ethnicity/race. |
| Two comments about language. This is not a survey about health outcomes. It is about sexual/reproductive health histories and use of/access to preventive services. There are no outcomes here. | Thank you, we agree that your suggested language is more accurate and have updated the title, abstract, manuscript body and table titles to reflect the focus on histories and access to health services rather than outcomes. |
| Also, I would strongly suggest that the results do much more than “underscore the need for routine and systematic data collection” as written in your Abstract. They underscore the need for the health and corrections systems work together to assure that incarcerated women have access to appropriate and recommended preventive services! What else is the point of the research data collection???  Please strengthen the language of your conclusion. | Thank you for the suggestion. We agree and have strengthened our language in the abstract (lines 61-63). |
| Reviewer 2 |  |
| Thank you for this pilot survey study of people experiencing incarceration in Canada and their access to services. This study is extremely important and highlights papers from the United States on challenges to accessing GYN healthcare services in the carceral setting. | Thank you. |
| There are several papers on GYN outcomes in carceral settings in the US. Specifically including those and their highlighting of social/cultural/historic barriers worsening outcomes for specific vulnerable populations as a part of the introduction would highlight the parallels you note for indigenous and gender diverse folks. There are also several papers on indigenous health outcomes that offer reasons for that variation specifically in Canada. I think the introduction can be expanded to explain why you created such a broad survey. Some information about the carceral system for women in Canada would be helpful to put this specific provincial prison in context. Some grammar change for flow would be helpful. | We have added citations to US studies on lines 96-99, which do provide valuable context to the barriers to accessing reproductive health services in prison settings.  With respect to Indigenous health in prisons, we feel it is important to focus on Canadian literature given the unique past and current experience of colonialism in Canada. We have added more context to the over-incarceration of Indigenous women and specific barriers to accessing reproductive health services on lines 114-117.  We have added further context on the Canadian prison system under the subheading ‘Setting’ on lines 144-149.  Additionally, we have edited for grammar and flow throughout the introduction. |
| Methods- Very clear and direct. Would you be able to share your survey instrument? | Apologies as we cited the instrument being available in Appendix A but did not upload the supplementary file. We have uploaded the instrument to supplementary files. |
| I would remove the "NO: categories from your questions. Just list the "YES" and "don't know" so that we can reduce the size of tables and streamline appearance. | Thank you for this suggestion. We considered it carefully, however we decided to leave the NO categories in the table as we feel it is important to accurately represent the options provided to participants when filling out the survey. |
| Consider not reporting data <5. | Thank you for this suggestion. We considered it carefully along with the prior comment. However, we feel it is important to accurately represent the options provided to participants when filling out the survey. |
| Pap tests can happen every 5 years--why does q3 years raise concern? | We are reporting based on national Canadian guidelines, which recommended a Pap test every three years, which is cited in the paper on line 259. |
| Given the majority of indigenous identifying folks, is it possible to break some data down to specifically look at indigenous versus general population responses to see if there is a difference in experience? Do their qualitative responses add any details explaining their experience? I only ask because one of the major conclusions of this paper is the need for culturally sensitive care. I'm not sure if its lack of basic access to resources/racism or culturally concordant care that's driving these outcomes and I'm also not convinced we can make that conclusion with the current data. | Thank you for the suggestion. We considered it carefully however due to the small sample size and slightly less than half of participants identifying as Indigenous, we are not able to break down the data to look at Indigenous vs. general population responses. We did re-review qualitative responses and did not identify any details specific to Indigenous participants' experiences. We do not claim that lack of culturally concordant care is driving these disparities, and agree with you that this claim cannot be made with this survey data. We do however feel that is appropriate to call for future attention to the needs of Indigenous people due to their over-representation in the prison system in Canada, as we do on line 297-300 and line 333-335. |
| I think a paragraph on recommended next steps to address the limitations of this paper may be useful. | We have added future recommendations to address study limitations on lines 316-324: *:In order to address some of these limitations, we recommend routine and systematic data collection on reproductive, gynecological and breast health history and outcomes in this population across multiple jurisdictions. This data would allow for better understanding of reproductive health histories and status for services amongst provincially incarcerated women broadly and would help to identify region-specific needs and opportunities for the development of healthcare programming and policy. Future research could also seek the perspectives of prison healthcare staff and leadership in order to better understand the current availability and provision of services, and to identify resource and knowledge needs with respect to reproductive, gynecological and breast health.* |
| Is there a way to get data on other provincial prisons in Canada to generally discuss reproductive access and its challenges? Do all prisons have access to a gynecologist? Is there any way to get this information? It may be that this data does not exist, but perhaps highlighting what next steps would be needed (again) would help clarify some of the statements in your conclusion. | Data on reproductive health outcomes in other provincial prisons is not available. We previously conducted this survey in Atlantic Canada, which we cite in the discussion on line 270 to highlight how both surveys identified high lifetime rates of unintended pregnancy and abortion, as well as high rates of housing precarity and low income. We agree that additional data is needed and have included this in the paragraph on recommended next steps in response to the above comment. |
| Please include recall bias in your limitations. | We have added this as a limitation on lines 313-315. |
